# Advancing Person-Centered Dementia Care in Long-Term Care Settings: Global Perspectives From Five Countries

**DOI:** 10.1093/geroni/igaf122.692

**Published:** 2025-12-31

**Authors:** Jing Wang, Kirsten Corazzini, Edward Miller

## Abstract

As the global population ages, long-term care (LTC) systems across diverse cultural and economic contexts face mounting challenges in providing person-centered dementia care (PCDC). This symposium brings together five studies from five countries, highlighting innovative strategies, workforce-driven facilitation, quality-of-life conceptualizations, behavioral symptom management, and technology-driven interventions in LTC settings. By examining experiences across different healthcare systems, this session offers insights into strengthening dementia care through interdisciplinary, culturally responsive, and sustainable approaches. The first presentation (United States) explores resilience-based strategies that enable LTC staff to uphold PCDC despite resource limitations, identifying organizational, interpersonal, and individual strengths that foster adaptability. The second study (Canada) challenges traditional facilitation frameworks by demonstrating how frontline workers in nursing homes informally drive care innovations, filling gaps between leadership initiatives and direct caregiving. The third presentation (Netherlands) investigates how nursing home administrators conceptualize and promote quality of life (QoL) for persons with dementia, revealing tensions between individualized and community-centered approaches. The fourth study (China) examines agitation in older LTC residents with cognitive impairment, identifying key individual, family, staff, and facility-level factors that contribute to behavioral symptoms. The final presentation (Singapore) evaluates an interdisciplinary telemedicine program aimed at reducing avoidable emergency department visits among nursing home residents, demonstrating how technology can optimize acute care management. Together, these studies provide a comprehensive, cross-national perspective on sustaining and enhancing person-centered dementia care in LTC. They underscore the need for workforce support, culturally informed care strategies, innovative facilitation models, and scalable technology-enabled solutions to improve dementia care across diverse healthcare settings. Common Data Elements for International Research in Residential Long-Term Care Interest Group Sponsored Symposium

